# AI Data-Driven Personalisation and Disability Inclusion

**DOI:** 10.3389/frai.2020.571955

**Published:** 2021-01-18

**Authors:** Mike Wald

**Affiliations:** University of Southampton, Southampton, United Kingdom

**Keywords:** personalisation, classification‐, localisation, artificial intelligence, disability

## Abstract

This study aims to help people working in the field of AI understand some of the unique issues regarding disabled people and examines the relationship between the terms “Personalisation” and “Classification” with regard to disability inclusion. Classification using big data struggles to cope with the individual uniqueness of disabled people, and whereas developers tend to design for the majority so ignoring outliers, designing for edge cases would be a more inclusive approach. Other issues that are discussed in the study include personalising mobile technology accessibility settings with interoperable profiles to allow ubiquitous accessibility; the ethics of using genetic data-driven personalisation to ensure babies are not born with disabilities; the importance of including disabled people in decisions to help understand AI implications; the relationship between localisation and personalisation as assistive technologies need localising in terms of language as well as culture; the ways in which AI could be used to create personalised symbols for people who find it difficult to communicate in speech or writing; and whether blind or visually impaired person will be permitted to “drive” an autonomous car. This study concludes by suggesting that the relationship between the terms “Personalisation” and “Classification” with regards to AI and disability inclusion is a very unique one because of the heterogeneity in contrast to the other protected characteristics and so needs unique solutions.

## Introduction

This study aims to help people working in the field of AI understand some of the issues regarding disabled people who are greatly disadvantaged in society in many ways.

The United Kingdom government states^1^ that there are over 11 million people with a limiting long-term illness, impairment, or disability, and the prevalence of disability rises with age (6% of children, 16% of working age adults, and 45% over state pension age). Compared to people who are not disabled, disabled people are substantially more likely to live in poverty, less likely to be employed, three times as likely not to have qualifications, and half as likely to hold a degree level qualification.

Artificial intelligence technologies, such as seeing AI^2^, are improving in their abilities to identify objects and faces. This application was created by a blind developer, and although such useful technologies are being developed by talented people with a deep knowledge and understanding of the needs of people with visual impairment, most technology developers do not have such a deep knowledge or understanding and do not learn about disability and accessibility on their university courses.

Data-driven personalisation normally implies the use of some sort of AI classification algorithm, and this study examines the relationship between the terms “Personalisation” and “Classification” with regard to disability inclusion. Classification using big data struggles to cope with the individual uniqueness of disabled people^3^, and whereas developers tend to design for the majority so ignoring outliers, designing for edge cases would be a more inclusive approach as these solutions will also work for the majority.

Since AI machine learning classification categorises people into groups and needs big data to do this, it struggles to cope with the individual uniqueness of disabled people. Of all the protected characteristics groups covered by the United Kingdom Equality Act^4^ (age, disability, gender reassignment, race, religion or belief, sex, sexual orientation, marriage and civil partnership, and pregnancy and maternity), disability is the most heterogeneous.

This study begins by examining definitions of personalisation and classification and discussing whether “group size” is the main factor.

It then presents two simple common examples (buying clothes and buying a pencil with a name on it) to clarify that “data driven personalisation” in the context of AI is normally taken to mean that the data have not been provided for that explicit purpose by the person. The examples also indicate how diversity (culture and disability) is often not adequately provided for in AI training datasets.

The next section examines some specific issues relating to the use of technologies by disabled people. The example of the difficulty of selecting the optimum accessibility setting on a mobile phone from the near infinite possibilities is described, and a possible solution is presented. The example of an autonomous vehicle is then provided to illustrate some of the ethical issues involved and also how not including disabled people in the training data could have disastrous consequences. Speech recognition is also provided as another example of how the unique requirements of disabled people may not be adequately catered for by standard AI solutions. The question whether localisation is “personalisation” for a cultural group is then discussed and illustrated through the example of the author’s work on developing Arabic symbols for Arabic people unable to communicate in speech or writing, The section ends with a brief discussion of the potential of neurosymbolic AI that integrates probabilistic machine learning with structured symbolic AI to help overcome many issues such as small datasets and explainability.

The review and discussion of relevant literature covers a wide range of issues concerning AI and disabled people.

The study finishes with a conclusion section that summarises the study’s arguments and identifies some of the remaining challenges.

## Relationship between Personalisation and Classification

This study will first examine the relationship between the terms “Personalisation” and “Classification.”

The Cambridge dictionary definitions^5^ are as follows:

Personalization^6^: “the process of making something suitable for the needs of a particular person”

Classification^7^: “the act or process of dividing things into groups according to their type”

This raises the issue of whether we can only think of classification as personalisation when there is just one member of a group or whether classification can be thought of as personalisation for every member of a group and whether the term personalisation should only be used for a maximum group size. The range of personalisation could be from a unique group of one through dividing everyone into many groups to the extreme of no personalisation where everyone gets the same and so is in just one group.

Data-driven personalisation also raises the issue of who originally created the data.

If the data used were originally created by the person who the data refer to can this be called “data driven personalization,” or for this to be the case, must the data be inferred from other data?

For example, considering classification and personalisation with regards to clothing, very large group classification could be into two groups based on gender, e.g., blue boy baby outfit/pink girl baby outfit; smaller group classification could be based on color or style or size (e.g., an “off the peg” suit); and personalised clothing could be a unique made to measure suit.

If somebody simply supplied the exact data of the details of color, style, or measurements for a made to measure suit then, although these data have driven the personalisation, I doubt this is what most people would refer to as “data driven personalisation.” I would suggest most people would rather think of “Data driven personalisation,” for example, suggesting suits based on those you have bought previously; suggesting suits based on purchases of those people who have also bought the suits you have bought previously; or estimating your preferences and measurements from photos of you.

However, for somebody with a physical disability, they may not be able to put on or take off standard clothing independently; may not fit any “off the peg” clothing; and may not fit any standard algorithms based on photos and so could be an “outlier” in any existing clothing related dataset and so not benefit from standard AI data-driven personalisation algorithms.

Let us also use as an example somebody buying a pencil with their name on it. There are various possibilities. They could select a pencil with their name already on it from a shop where there can only be a limited number of most popular names available. They could have their name printed to order with their name provided directly by themselves. They could have their name printed with their name provided indirectly (e.g., through data from Facebook if signed up through Facebook). A company could send an unsolicited promotional marketing free gift of pencil with printed name with name provided indirectly (e.g., through data obtained from their Facebook postings). Only the indirectly provided names would be considered “data driven personalization.” People from a nonnative culture would have a much lower chance of finding their name as one of the limited number of names available in the shop. A person with a disability might also require a nonstandard shaped pencil to help them be able to write.

The next section examines some specific issues relating to the use of technologies by disabled people.

## Technologies and Disabilities

There are many aspects of personalising technologies for a disabled person. They can have different strengths (e.g., visual, auditory, kinesthetic, dexterity, mobility, confidence, processing speed and attention, health, memory, technology skills, motivation, knowledge, and experience). There can be different tasks (e.g., reading and understanding information, writing, organisation and planning, communication, memory and recall, time, money, numeracy, and daily living). They can have access to different resources (e.g., financial, training, peer support, professional support, and technical support). They can be in different environments (e.g., workplace, study, daily life, accessibility constraints, security, and IT policies) and using different tools (text to speech and e-reading, word processing and proofing, graphical mapping and planning, reminders, speech recognition, calculators and mathematics, study support, alarms and environmental controls, wearable technologies, and communication devices).

Technologies can have many personalisation settings to accommodate the individual needs of disabled people, and the example of a mobile phone will be used to illustrate the issue of how the optimum settings can be chosen.

### Personalising a Mobile Phone

A disabled person can change the accessibility settings on their phone, but on the iPhone, for example, I have calculated that there are as many unique permutations of accessibility settings as there are atoms in the known Universe, and so, while it would be possible in theory for every person to create a unique personalised setting, it would be practically impossible for somebody to actually try all the possible permutations of settings out. Interoperable accessibility profiles would allow disabled people’s preferred settings to work on any system anywhere in the world, but since settings are not interoperable between different manufacturers’ devices, a person would have to set up every device they used. Some of these settings may be more important than others to a person (e.g., increasing the rate of speech (when using “text to speech” for speaking out text for people with reading difficulties) by 5% will not have as much effect as changing it by 20%), but having some automated systems to make these selections could speed up this personalisation process. For example, where there are a large range of settings such as speaking rate, the system could adaptively find the chosen setting using comparisons of pairs of settings and measuring just noticeable differences. For example, 5 possible settings of speaking rate from 1 to 5 could involve listening to 10 pairs of settings to compare them all, but an adaptive system could only involve listening to and comparing 3 pairs of rate settings using the following algorithm.

Listen to and compare 1 and 5, and if no preference, then 1 is the final selection and have listened to only 1 pair.

If preferred 5 over 1, then listen to and compare 5 with 3, and if no preference, then compare 3 (i.e., if no preference, we arbitrarily choose lowest setting and assume there would also be no preference with 4) with 2. If preferred 2 or no preference, then 2 is the final selection, and if preferred 3, then 3 is the final selection and have listened to only 3 pairs.

If preferred 1 over 5, then listen to 3, and if no preference, then compare 1 and 4. If preferred 1 to 4 or no preference, then 1 is the final selection. If preferred 4, then 4 is the final selection and have listened to only 3 pairs.

If preferred 1 over 3, then listen to 2. If no preference or 1 preferred, then 1 is the final selection. If preferred 2, then 2 is the final selection and have listened to only 3 pairs.

If preferred 5 over 3, then listen to 4, and if 4 preferred or if no preference, then 4 is the final selection. If 5 preferred, then 5 is the final selection and have listened to only 3 pairs.

There is a privacy issue whether the disability of somebody can be determined from the settings shared with 3rd parties. For example, if they have their screen reader turned on, then they are very probably visually impaired/blind.

It would be possible to infer accessibility settings using a recommender type system from people with similar disabilities as a starting point from which somebody could further personalise their system settings.

The next subsection uses the example of an autonomous vehicle to illustrate some of the ethical issues involved and also how not including disabled people in the training data could have disastrous consequences.

### Autonomous Vehicles

Autonomous vehicles issues include how they will make ethical decisions (e.g., avoid a child but kill an elderly person). Will there be one globally accepted ethical algorithm? Will each car manufacturer have their own ethical algorithm? Will the owner select from a choice of ethical algorithms? Will the car learn from how the owner drives and behaves and personalise an ethical algorithm from this? Will a blind or visually impaired person be permitted to “drive?”^8^ How will autonomous vehicles respond to disabled “pedestrians?” An example of the issue is that if a disabled person in a wheelchair cannot use their arms to push themselves along, they can use their legs to push themselves backwards and even possibly use a mirror to see where they are going. When the scenario of a disabled person in a wheelchair crossing the road was put into a self-driving car simulation, the car ran the simulated wheelchair user over as it misunderstood which way the person was crossing^9^. Developers tend to design for the majority ignoring outliers, whereas designing for edge cases would be a more inclusive approach. It is, therefore, also important to include disabled people in decisions to need to understand AI implications. Also, AI could be used to help wheelchair users independently control manual or electric wheelchairs or people with cognitive disabilities (e.g., dementia) travel or navigate independently.

The next subsection uses speech recognition as another example of how the unique requirements of disabled people may not be adequately catered for by standard AI solutions.

### Speech Recognition

Speech recognition can help people who have difficulty writing to use their voice to write. It can also assist people who have difficulty hearing by providing captions and transcripts. Speech recognition was originally personalised for each individual through extensive training by that individual on systems installed locally, but now, cloud-based speaker independent recognition is ubiquitous, and only one locally installed speaker dependant recognition software is commercially available^10^. There is little commercial benefit for companies to develop speech recognition, speech synthesis, or machine translation for minority languages. Standard speech recognition also does not work well for people with dysarthric speech and so needs a special system (Hawley et al., 2019). Using AI for lipreading has been shown to increase the accuracy of speech recognition and especially in noise^11^. The growing availability and reduction in cost of 3D cameras^12^ should help continue to improve accuracy. Many people have expressed concerns about “Deepfakes”^13^ where AI has, for example, been used to control people’s lip movements and speech to make them appear to say things they never said. Nobody, however, appears to have thought of using the same technology to make people more lipreadable. Automatic captions can indicate some nonspeech sounds (e.g., music, laughter, and applause^14^), and emotion detection from speech^15^ and faces^16^ is improving.

For people who will lose their voice due to disease, a personalised voice can be created before this occurs^17^.

The question whether localisation is “personalisation” for a cultural group is discussed and illustrated in the next subsection through the example of the author’s work on developing Arabic symbols for Arabic people unable to communicate in speech or writing.

### Localisation

Localisation can be defined as “the process of making a product or service more suitable for a particular country, area, etc.”^18^

Is localisation “personalisation” for a cultural group? Assistive technologies can need localising in terms of language as well as culture. We developed Arabic symbols for people who found it difficult to communicate in speech or writing because many western symbols were not culturally appropriate and also some cultural symbols did not exist^19^. These symbols were created by a graphic designer working with symbol users and so were expensive and time consuming to produce. We are currently investigating ways in which AI could be used to create symbols automatically from photographs.

To be able to select the required symbol from a hierarchical structured symbol board can take a long time (e.g., select foods at top level board, vegetables at next level board, and cauliflower from the vegetable board), and so, it would be more efficient to automatically select the required symbols based on the context (e.g., system knows user is in supermarket and knows their shopping list).

The final subsection gives a brief discussion of the potential of neurosymbolic AI that integrates probabilistic machine learning with structured symbolic AI to help overcome many issues such as small datasets and explainability.

### Neurosymbolic AI

Machine learning can use deep neural networks to develop probabilistic models from large training datasets without having prior knowledge of the knowledge structure of the data. This has, for example, allowed the development of speech recognition and machine translation systems that do not need to be provided with a model of language structure.

Symbolic AI methods can use logic-based structured semantic conceptual knowledge representation and reasoning from ontologies or knowledge graphs to help create rules that do not require the large training datasets needed by many machine learning methods.

Neurosymbolic AI^20^ is an approach that tries to integrate machine learning approaches with symbolic methods to gain the combined benefits of both approaches (e.g., where large datasets are not available and perhaps where less computing power is available and also to help provide explainable or verifiable AI).

While this can help in overcoming the limited information about disabled individuals available in machine learning training datasets, it can only “broadly” categorise disabled individuals in terms of their disabilities rather than personalise a disabled individual in terms of their unique abilities and disabilities.

This approach could, however, for example, help reduce the number of possible accessibility settings in their mobile phone; a disabled individual would need to select from to find their personalised optimum setting.

Mao et al. (2019) presented a method that jointly learns visual concepts, words, and sentences from images, questions, and answers and suggested applying neurosymbolic learning frameworks as a future work toward automatic learning in complex interactive environments. Although not discussed in the study, this would appear to have particular potential for assisting blind people navigating and interpreting their environment.

Kursuncu et al. (2020) proposed a learning framework that infuses domain knowledge within the neural networks unlike previous approaches that utilized knowledge outside neural attention models to provide “better generalizability, reduction in bias and false alarms, disambiguation, less reliance on large data, explainability, reliability, and robustness, to the real world applications.”

Besold et al. (2017) reviewed ideas on neurosymbolic learning and reasoning and outline some of the technical challenges while acknowledging “knowledge about these issues is only limited and many questions still have to be asked and answered” with impact “in many areas including the web, intelligent applications and tools, and security.”

Arabshahi et al. (2020) inferred missing presumptions through reasoning to discover commonsense knowledge from if-then-because statements from a human-derived dataset.

Readers wishing to know more about the many current technical approaches to neurosymbolic AI may find the recent presentation by Alexander Gray (IBM Research) “A recent review of Neuro-Symbolic AI: Overview and Open Questions” of interest^21^

## Review and Discussion of Relevant Literature

This section discusses some published studies regarding a range of issues concerning AI and disabled people.

Draffan et al. (2019a) discussed how data collections are not often inclusive or algorithms transparent. They presented a roadmap for digital accessibility research and development using AI to support those with disabilities with examples where strategies can help prevent barriers to inclusion. Their extensive literature review showed how “disability” was wrongly considered as a homogeneous concept and inclusion did not consider accessibility or design for all or equity of access. They concluded that algorithms needed to be designed for inclusion by removing bias and ensuring fairness to achieve enhanced digital accessibility.

Datasets used to train machine learning algorithms can exclude or underrepresent disabled people and so discriminate against them (e.g., education, employment, and credit) (Gilligan, 2019). A loan may be refused because the applicant is wrongly classified whether due to ignorance, motivated by good intentions with respect to privacy or safety or ethical concerns, or no better dataset exists. Preprocessing techniques such as oversampling and undersampling can help equalise the size of the classes, but it would be better to have inclusive datasets for underrepresented groups respecting ethics, privacy, and safety.

“AI bias” can marginalize disabled people by classifying them as outliers affecting fair access to important services (e.g., health insurance and credit). The IBM Fairness 360 Open Source Toolkit’s algorithms^22^ claim to “examine, report and mitigate discrimination and bias in machine learning models.” Zimmerman et al. (2019) studied the effect of AIF360 on the accuracy of gender recognition for face images of persons with and without Down syndrome (DS) in the proportion of persons with DS in the German population (0.1%). They found the AIF360 toolkit has the potential for mitigation of AI bias, but a larger sample is needed to confirm this.

Wolters (2019) examined the extent to which ergonomic and accessibility issues are acknowledged and discussed in the literature but found that research studies only consider eHealth solutions for chronic pain management and not ergonomic or accessibility aspects and concluded that this needed to be undertaken before leveraging AI meaningfully to address them.

Individuals with complex communication needs can use symbols with text translations, but data are scarce, and conversions are fraught with complications due to the different types of linguistic concepts, imagery, and language and limited harmonization or standardization, and so, users find it hard to access suitable personalised or localized symbols. Draffan et al. (2019b) examined how symbol sets can be linked with multilingual options using AI image recognition to improve outcomes by automatically creating a more diverse range of symbols based on transforming photos.

Potter et al. (2019) identified four pitfalls in the use of deep learning for personalisation of assistive technology in order to help allocate scant resources to benefit end users: fallacies that there is “true” knowledge inherent in data; mistakes that derive from ignorance of the limitations of methods; constraints of human commerce; and failings from incorrect, ill-considered, or improper use of AI.

Another issue of data-driven personalisation is the ethics of AI for “eugenics” or “curing” neurodiversity (e.g., biomarkers for autism) or disability. It is offensive to people with autism to see this as something people should aim for, and so, individuals with autism and their families need to be treated with respect and understanding (Walsh et al., 2011). Hens et al. (2019) discussed “whether autism is a disorder to be treated or an identity to be respected.”

The power of AI deep learning to search the human genome for mutations and prediction of autism or other conditions (Zhou et al., 2019) increases the possibility of data-driven “personalisation” for parents to ensure their babies are born without disabilities.

Johnston (2005) argued that “the premise that deafness is not a disability of some sort is false and thus the claim that genetic selection against deafness is unethical is untenable.”

A deaf lesbian couple turned to a friend with five generations of deafness in his family after being turned away by a sperm bank which told them that donors with disabilities were screened out ^23^.

Clause 14/4/9 of the Human Fertilisation and Embryology (HFE) bill^24^ blocks any attempt by couples to use modern medical techniques to ensure their children are deaf as it states that “Persons or embryos that are known to have a gene, chromosome or mitochondrion abnormality involving a significant risk that a person with the abnormality will have or develop a serious physical or mental disability, a serious illness or any other serious medical condition must not be preferred to those that are not known to have such an abnormality.”

Fayemi (2014) discussed the need for “prenatal genetic testing, as well as abortion of foetuses with a high risk of the autism mutation.”

Johannessen et al. (2017) discussed how “Adults with ASD fear that people with ASD traits eventually will be eliminated through prenatal testing and selective abortion” and that “professionals believe that genetic testing could improve the possibility for early intervention” and reported the results of their study of parent members of the Norwegian Autism Society, 76% of whom would undergo clinical genetic testing if it would improve the possibilities for early interventions.

## Conclusion

This study will hopefully have helped people working in the field of AI understand some of the issues regarding disabled people.

This study has suggested that the relationship between the terms “Personalisation” and “Classification” with regards to AI and disability inclusion is a very unique one because of the heterogeneity in contrast to the other protected characteristics and so needs unique solutions.

This can, for example, result in assistive technologies developed for a broad category of disability (e.g., visually impaired people or hearing impaired people) not being appropriate or the optimum for a particular individual with a specific unique visual impairment or hearing impairment as well as perhaps other disabilities.

Issues that have been discussed in this study include personalising mobile technology accessibility settings with interoperable profiles to allow ubiquitous accessibility, the ethics of using genetic data-driven personalisation to ensure babies are not born with disabilities, the importance of including disabled people in decisions to help understand AI implications, the relationship between localisation and personalisation as assistive technologies need localising in terms of language as well as culture, the ways in which AI could be used to create personalised symbols for people who find it difficult to communicate in speech or writing, whether blind or visually impaired person will be permitted to “drive” an autonomous car, and how neurosymbolic AI can help reduce the number of possible accessibility settings in a disabled individual would need to select from to find their personalised optimum setting.

Classification using big data struggles to cope with the individual uniqueness of disabled people^25^; whereas developers tend to design for the majority so ignoring outliers, designing for edge cases would be a more inclusive approach as these solutions will also work for the majority. It is, therefore, important for AI developers to involve disabled people when developing AI systems.

Technology that accommodates the needs of disabled people can also often better meet the needs of nondisabled people (e.g., captions for deaf people can help everyone when the sound is not available such as in airport lounges).

There are still many challenges for AI to support disabled people. For example, automatic audio description of videos requires reasoning and understanding subtle meanings and context to identify what visual information is important (e.g., if a person leaves a room, is it important to know they did not hear what was said after they left?), and while AI can help provide automatic sign language translation of captions using human video clips or avatars, the quality of translation for a visual language is not currently as good as translations between written languages which have vast amounts of data available for training the AI systems.

## Data Availability Statement

The original contributions presented in the study are included in the article/Supplementary Material, further inquiries can be directed to the corresponding author.

## Author contributions

The author confirms being the sole contributor of this work and has approved it for publication.

## Funding

This work was supported by The Alan Turing Institute under the EPSRC grant EP/N510129/1.

## Conflict of Interest

The author declares that the research was conducted in the absence of any commercial or financial relationships that could be construed as a potential conflict of interest.

## Footnotes

1
https://www.gov.uk/government/publications/disability-facts-and-figures/disability-facts-and-figures

2
https://www.microsoft.com/en-us/seeing-ai

3
https://venturebeat.com/2018/12/03/how-to-tackle-ai-bias-for-people-with-disabilities/

4
https://www.legislation.gov.uk/ukpga/2010/15/contents

5
https://dictionary.cambridge.org/

6
https://dictionary.cambridge.org/dictionary/english/personalize

7
https://dictionary.cambridge.org/dictionary/english/clarification

8
https://www.eng.ufl.edu/newengineer/news/transforming-the-autonomous-vehicle-experience-for-the-blind/

9
http://openresearch.ocadu.ca/id/eprint/1880/1/Treviranus_TransportationTalk_2017.pdf

10
https://www.nuance.com/dragon.html

11
https://arxiv.org/abs/1611.01599

12
https://www.apple.com/uk/iphone-xs/cameras/

13
https://www.theguardian.com/technology/2020/jan/13/what-are-deepfakes-and-how-can-you-spot-them

14
https://youtube-eng.googleblog.com/2017/03/visualizing-sound-effects.html.

15
https://s3-us-west-1.amazonaws.com/vocals22/VENEC/plot_sounds_auto_expdimem.html.

16
https://www.paralleldots.com/facial-emotion

17
https://www.acapela-group.com/voices/voice-banking/

18
https://dictionary.cambridge.org/dictionary/english/localisation

19
https://globalsymbols.com/symbolsets/tawasol

20
https://daselab.cs.ksu.edu/content/neuro-symbolic-integration-and-explainable-artificial-intelligence

21
https://researcher.watson.ibm.com/researcher/view_group.php?id=10518

22
https://aif360.mybluemix.net/

23
https://www.theguardian.com/world/2002/apr/08/davidteather

24
http://www.legislation.gov.uk/ukpga/2008/22/section/14

25
https://venturebeat.com/2018/12/03/how-to-tackle-ai-bias-for-people-with-disabilities/
